# Identification of novel esterase-active enzymes from hot environments by use of the host bacterium *Thermus thermophilus*

**DOI:** 10.3389/fmicb.2015.00275

**Published:** 2015-04-08

**Authors:** Benedikt Leis, Angel Angelov, Markus Mientus, Haijuan Li, Vu T. T. Pham, Benjamin Lauinger, Patrick Bongen, Jörg Pietruszka, Luís G. Gonçalves, Helena Santos, Wolfgang Liebl

**Affiliations:** ^1^Department of Microbiology, Technische Universität MünchenFreising, Germany; ^2^Research Center Juelich, Institute of Bioorganic Chemistry, Heinrich-Heine-Universität DüsseldorfJuelich, Germany; ^3^Instituto de Tecnologia Química e Biológica, Universidade Nova de LisboaOeiras, Portugal

**Keywords:** *Thermus thermophiles*, novel screening host, functional metagenomics, comparative screening, novel metagenomic esterases

## Abstract

Functional metagenomic screening strategies, which are independent of known sequence information, can lead to the identification of truly novel genes and enzymes. Since *E. coli* has been used exhaustively for this purpose as a host, it is important to establish alternative expression hosts and to use them for functional metagenomic screening for new enzymes. In this study we show that *Thermus thermophilus* HB27 is an excellent screening host and can be used as an alternative provider of truly novel biocatalysts. In a previous study we constructed mutant strain BL03 with multiple markerless deletions in genes for major extra- and intracellular lipolytic activities. This esterase-diminished strain was no longer able to grow on defined minimal medium supplemented with tributyrin as the sole carbon source and could be used as a host to screen for metagenomic DNA fragments that could complement growth on tributyrin. Several thousand single fosmid clones from thermophilic metagenomic libraries from heated compost and hot spring water samples were subjected to a comparative screening for esterase activity in both *T. thermophilus* strain BL03 and *E. coli* EPI300. We scored a greater number of active esterase clones in the thermophilic bacterium than in the mesophilic *E. coli*. From several thousand functionally screened clones only two thermostable α/β-fold hydrolase enzymes with high amino acid sequence similarity to already characterized enzymes were identifiable in *E. coli*. In contrast, five further fosmids were found that conferred lipolytic activities in *T. thermophilus* only. Four open reading frames (ORFs) were found which did not share significant similarity to known esterase enzymes but contained the conserved GXSXG motif regularly found in lipolytic enzymes. Two of the genes were expressed in both hosts and the novel thermophilic esterases, which based on their primary structures could not be assigned to known esterase or lipase families, were purified and preliminarily characterized. Our work underscores the benefit of using additional screening hosts other than *E. coli* for the identification of novel biocatalysts with industrial relevance.

## Introduction

As only a small fraction of all microorganisms from environmental samples can be isolated and studied by classic microbiological methods, culture-independent strategies, such as metagenomics, have been employed to overcome these restrictions (Torsvik et al., 1990; Amann et al., 1995; Handelsman et al., 1998). The cloning and analysis of environmental DNA has led to the identification of various new genes for valuable enzymes (for a recent review see Leis et al., 2013). Sequence-based metagenomics strategies have been successful in many cases, but sequences without similarity to known genes cannot be identified using this approach (Steele et al., 2009; Liebl, 2011; Liebl et al., 2014). Function-based strategies on the other hand rely on the direct detection of functional gene products via diverse screening setups. Until today, *E. coli* is the most commonly used bacterial host in functional metagenomics. Since only a fraction of the genes from random (meta)genomic gene libraries are expressed (Gabor et al., 2004; Sorek et al., 2007), the identification of truly novel genes from metagenomes is hampered when a single host is used (Jenney and Adams, 2008). Other hosts than *E. coli* (e.g., diverse Proteobacteria, Actinobacteria, and eukaryotic fungi) have been shown to be good alternatives and new broad-range expression vectors have been developed for use in functional metagenomics (Courtois et al., 2003; Martinez et al., 2004; Aakvik et al., 2009; Craig et al., 2010; Damon et al., 2011; Kakirde et al., 2011; Kellner et al., 2011; Parachin and Gorwa-Grauslund, 2011; Liebl et al., 2014).

In previous work we have shown that *Thermus thermophilus* HB27 can be used for screening of genomic libraries using the shuttle fosmid pCT3FK. These studies resulted in the identification of several genes that would not have been found by using the mesophilic *E. coli* host alone (Angelov et al., 2009; Leis et al., 2013).

In this study we present the generation of large-insert metagenomic libraries and their subsequent screening for lipolytic activities in *E. coli* and *T. thermophilus* HB27. For the screenings in *T. thermophilus*, a multiple clean deletion mutant was used (termed BL03) which lacks several characterized extracellular and putative esterase encoding genes (Leis et al., 2014). The knockout strain had a substantially lower esterase activity than the wild type and the deletions abolished its ability to grow on SH minimal medium supplemented with tributyrin. Therefore, we decided to use this esterase-deficient strain in a high-throughput screening and selection setup where complementation of growth on tributyrin minimal medium by cloned heterologous metagenomic genes served to select for new environmental genes encoding tributyrin-cleaving esterases.

## Materials and methods

### Bacterial strains and growth conditions

Transformation and propagation of recombinant plasmids were performed using *Escherichia coli* XL1-Blue, XL10 GOLD® ultracompetent cells (Stratagene, La Jolla, USA) and DH10B (Invitrogen, Carlsbad, USA). *E. coli* EPI300-T1® (Epicentre, Madison, USA) was used for the generation and screening of metagenomic libraries cloned in pCT3FK large-insert fosmids (Angelov et al., 2009) based on pCC1FOS) upon addition of fosmid autoinduction solution (Epicentre). *E. coli* strain BL21(DE3) was used for the expression of target proteins in pET21a(+) (Novagen, Merck KGaA, Darmstadt, Germany). Bacterial cultures were grown in lysogeny broth (LB) or on LB agar plates supplemented with ampicillin (100 μg/ml), kanamycin (20 μg/ml) and chloramphenicol (12.5 μg/ml) as necessary. For *T. thermophilus* HB27 (DSMZ 7039) and *T. thermophilus* BL03 (genotype ΔTT_P0042, ΔTT_C0340-1, ΔTT_C0904, ΔTT_C1787), culturing and transformations were done as described in Angelov et al. (2009), using TB complex medium (8 g/l trypticase peptone, 4 g/l yeast extract, and 3 g/l NaCl) (Ramírez-Arcos et al., 1998) and SH minimal medium (Leis et al., 2014) with high-carbonate mineral water Aqua Purania (TSI, Zeven, Germany) at pH 7.5. Cells were cultured at 70°C or at 60°C when using kanamycin (final concentration of 20 μg/ml) for the propagation of transformants. For the growth selection and complementation assay, bacterial suspensions from overnight-grown liquid cultures were washed three times in 50 mM phosphate buffer (pH 7.5) and adjusted to equal optical densities before spotting 5 or 10 μl on complex and minimal medium. For screening and selection purposes on solid medium, tributyrin with a final concentration of 1.0% (v/v) was emulsified with an Ultra-Turrax homogenizer (IKA, Staufen, Germany) immediately before autoclaving of the medium.

### Generation of a large-insert fosmid library and functional screenings

Water, sediment and biofilms were sampled from hot springs in the town of Furnas Azores, Portugal (37°46′21.78″N 25°18′14.673″W). Compost heap samples were collected from a composting plant (Bioenergiezentrum GmbH, Göttingen, Germany). After separation of microorganisms from the samples' matrices (filtration and scraping off cells from organic matter), the metagenomic DNA was prepared using the PowerSoil, PowerMax Soil and UltraClean Fecal DNA Isolation Kit (MO BIO, Carlsbad, USA). The generation of a large-insert DNA library was done using the pCC1FOS-derived pCT3FK *T. thermophilus*/*E. coli* shuttle fosmid (Angelov et al., 2009) according to the instructions of the pCC1FOS Fosmid Library Production Kit (Epicentre, Madison, USA). Arrayed clones from *E. coli* were cultured in 96-well microtiter plate format. To overcome labor-intensive transfer of single fosmid clones from *E. coli* to *T. thermophilus*, a high-throughput screening strategy was chosen based on growth complementation (Leis et al., 2014). Therefore, each pool of 96 single fosmids from *E. coli* was transferred in the screening strain *T. thermophilus* BL03 by a single transformation reaction. The transformants of each reaction were grown on TB plates containing kanamycin as antibiotic. After 2 days of incubation at 60°C, colonies were suspended from the plates and washed three times using 50 mM phosphate buffer (pH 7.5). The washed cell suspensions (each comprising a pool of 96 *T. thermophilus* transformants obtained by transformation of strain BL03 with pooled fosmids from 96 *E. coli* clones) were spotted onto SH minimal medium containing tributyrin (1% v/v) and kanamycin. Candidate clone pools were able to grow on tributyrin due to heterologous growth complementation after 2–3 days of incubation at 60°C. In order to determine single clone(s) whose fosmid-derived inserts conferred tributyrase activity within each positive pool, *T. thermophilus* BL03 was transformed individually with each of the 96 *E. coli* fosmids in deep well plate format as described before (Angelov et al., 2009). Suspensions of the transformants were washed several times and spotted on minimal medium containing tributyrin. *T. thermophilus* clones with candidate fosmid inserts grew after several days of incubation at 60°C. The stability of each phenotype was assessed by repeated streaking and growth of the clones on SH with tributyrin.

Each candidate clone was analyzed for its ability to hydrolyze the substrate in comparison to the control containing only the empty fosmid DNA. The corresponding halo formation was calculated as the area of the clearing zone without the area of the corresponding colony size according to the following equation:

$$\begin{array}{l} \text{Halo area formed}=\\ \;[\text{diameter}{\left(\text{halo}\right)}^{\text{2}}-\text{diameter}{\left(\text{colony}\right)}^{\text{2}}]\times \text{0}.\text{25}\times \pi \end{array}$$

### Sequencing and *in silico* analysis of sequence data

Fosmids conferring significant halo formation were sequenced using Roche's GS-FLX series 454 pyrosequencer at the Göttingen Genomics laboratory (G_2_L) and LGC Genomics (Berlin, Germany). For phylogenetic analysis, bacterial and archaeal 16S rDNA sequences were amplified by PCR using degenerate primer pairs 616Valt, 100K, AC165, and AC1601, respectively (Supplementary Table S1). Phylogenetic analysis was done using ARB (Ludwig et al., 2004) and the Ribosomal Database Project II (RDP-II) server (Cole et al., 2009). Assembled contig sequences were analyzed *in silico* using SEED (Overbeek et al., 2005). BlastP (Altschul et al., 1990), conserved domain database (CDD, Marchler-Bauer et al., 2011), and the Pfam database (Punta et al., 2012) were used for similarity searches and annotation. The presence of predicted signal peptides was analyzed with the SignalP server (Petersen et al., 2011). Regular expression algorithm was used to unravel potential esterase candidate genes by GXSXG conserved motif searches. For the generation of multiple sequence alignments and similarity tree reconstruction, representative protein sequences from classified esterases/lipases (Chow et al., 2012) were used. Tree calculations based on multiple sequence alignments were performed with the T-REX webserver (Boc et al., 2012) and the resulting Newick-formatted data was visualized with MEGA Version 5.2 (Tamura et al., 2011).

### Cloning, expression, and purification of metagenomic esterases

Candidate genes were PCR amplified with Phusion DNA polymerase (ThermoFisher Scientific, Waltham, USA) using primers listed in Supplementary Table S1 (oligonucleotides EstA2_for and EstA2_rev for cloning EstA2, EstB1_for and EstB1_rev for EstB1, respectively). The PCR products were cloned using a CloneJET vector (Fermentas) backbone. Insertion of the polyhistidine (6xHis) sequence was performed by the Change-IT Multiple Mutation Site Directed Mutagenesis Kit (Affymetrix, Santa Clara, USA) with 5′-phosphorylated oligonucleotides (EstA2_PHO_F and EstB1_PHO_F) according to the manufacturer's instructions. Metagenomic esterase-encoding genes (EstA2 and EstB1) were cloned in pET21a(+) (Novagen) using *Nde*I and *Nco*I, and the same strategy was used for cloning in the *T. thermophilus* vector pMK18 (de Grado et al., 1999) by isothermal DNA assembly using the Gibson Assembly Master Mix (New England Biolabs, Ipswich, USA) with vector primers pMK18_RBS_for and pMK18_RBS_rev. The insert was amplified with Est_rev and corresponding forward primers of the esterases. For expression, *E. coli* BL21(DE3) Star cultures (250 ml) were grown in Erlenmeyer flasks to mid-log phase (absorbance at 600 nm ranging from 0.6 to 0.7) and induced with isopropyl-β-D-thiogalactopyranoside (IPTG) at a final concentration of 1.0 mM. After 4 h at 37°C, the cultures were harvested by centrifugation and disrupted by sonication (Hielscher Ultrasonics GmbH, Teltow, Germany). After removal of the cell debris, the lysate was subjected to heat treatment (at least 60°C for 20 min). The supernatant containing the thermostable and soluble protein of interest was purified using Protino Ni-IDA 2000 protein purification system (Macherey-Nagel) according to the manufacturer's instructions. Protein separation and purity was determined with sodium dodecyl sulfate polyacrylamide gel electrophoresis (SDS-PAGE) based on Laemmli (1970).

### Enzyme assays

*E. coli* and *T. thermophilus* cells from 5 ml overnight grown cultures were harvested by centrifugation and washed twice in 50 mM Tris-HCl (pH 7.5). All steps were performed on ice. Cells were resuspended in half volume of Tris buffer and disrupted by sonication until the turbid suspensions became clear. Cell debris was removed by centrifugation (21,000 × g, 10 min at 4°C) and supernatants were used for protein quantification and measurement of intracellular lipolytic activities. The *para*-nitrophenyl (*p*NP) assays were performed according to Leis et al. (2014) by using 0.1 ml of crude lysates incubated in 50 mM Tris-HCl (pH 7.5) buffer with 1.25 mM *p*NP-caprylate, -laurate, and palmitate at 60°C. After stopping the reaction with 0.25 ml of 2 M sodium carbonate and incubation on ice for 15 min, the mixture was centrifuged at 21,000 × g for 5 min at 4°C. Esterase activity measurements for purified enzyme preparations were performed using 1.25 mM final concentrations of different *para*-nitrophenyl-substrates (*p*NP-propionate (C_3_), -butyrate (C_4_), -valerate (C_5_), -caproate (C_6_), -caprylate (C_8_), caprate (C_10_), -laurate (C_12_), -myristate (C_14_), and -palmitate (C_16_), all obtained from Sigma-Aldrich). The influence of the additives NaCl, KCl, CaCl_2_, MgCl_2_ (salts), and inhibitor EDTA (chelating agent) and phenylmethylsulfonylfluorid (PMSF), an agent known to inhibit serine/cysteine proteases as well as acetylcholinesterases, was tested at final concentrations of 1 mM. Other *p*NP-substrates were derived by esterification with 2-methyldecanoate (Reetz et al., 1997; Franken et al., 2010; Wu et al., 2013), Ibuprofen, Naproxen (Reetz et al., 2010; Sandström et al., 2012), 6-methyl-2-(*ortho-tolyl)hept-5-enoate* (Gaich and Mulzer, 2009), and indancarboxylic acid (Pietruszka et al., 2009) and the activity assays were performed at 60, 70, and 80°C under the conditions reported by López et al. (2014) and Chow et al. (2012).

One unit of specific enzymatic activity was defined as 1.0 μmol of *para*-nitrophenol per minute and mg protein released from the substrate. All measurements were performed at least in duplicates (*n* = 2).

### Synthesis of assay substrates—representative procedure

Synthesis of 4-nitrophenyl 6-methyl-2-(*ortho*-tolyl)hept-5-enoate: 4-Nitrophenol (538 mg, 3.87 mmol), 4-dimethylaminopyridine (47.3 mg, 387 μmol), and dicyclohexylcarbodiimide (798 mg, 3.87 mmol) were added to a solution of 6-methyl-2-(*ortho*-tolyl)hept-5-enoic acid (900 mg, 3.87 mmol) in 25 mL of dichloromethane under nitrogen. The mixture was stirred at room temperature. After 24 h the precipitate was filtered off and the solvent was removed under reduced pressure. The residue was purified by flash chromatography (petroleum ether/ethyl acetate, 99/1) to give the product as a yellow oil (1.07 g, 3.03 mmol, 78%). ^1^H-NMR (600 MHz, CDCl_3_,): *δ*_H_ [ppm] = 1.55 (s, 3 H, 1-H), 1.71 (s, 3 H, 2-H), 1.88 [dddd, ^2^*J*_(6a,6b)_ = 13.7 Hz, ^3^*J*_(6a,5b)_ =7.2 Hz, ^3^*J*_(6a,5a)_ = 7.2 Hz, ^3^*J*_(6a,7)_ = 6.9 Hz, 1 H, 6-H_a_], 2.07 [dd,^3^*J*_(5,6)_ = 7.2 Hz, ^3^*J*_(5,4)_ = 7.2 Hz, 2 H, 5-H], 2.26 [dddd, ^3^*J*_(6b,7)_ = 6.7 Hz, ^3^*J*_(6b,5a)_ = 7.2 Hz, ^3^*J*_(6b,5b)_ = 7.2 Hz, ^2^*J*_(6b,6a)_ = 13.6 Hz, 1 H, 6-H_b_], 2.44 (s, 3 H, arom.-C*H*_3_), 4.12 [dd, ^3^*J*_(7,6a)_ = 6.8 Hz, ^3^*J*_(7,6b)_ = 6.7 Hz, 1 H, 7-H], 5.14 [t, ^3^*J*_(4,5)_ = 7.2 Hz, 1 H, 4-H], 7.15- (m_c_, 2 H, arom.-C*H*), 7.18–7.25 (m, 3 H, arom.-C*H*), 7.35 (m_c_, 1 H, arom.-C*H*), 8.22 (m_c_, 2 H, arom.-C*H*). ^13^C-NMR (151 MHz, CDCl_3_): *δ*_C_ [ppm] = 17.7 (C-1), 19.8 (arom.-C*C*H_3_), 25.7 (C-5), 25.8 (C-2), 32.8 (C-6), 46.3 (C-7), 122.3 (arom.-*C*H), 122.3 (arom.-*C*H), 123.1 (C-4), 125.1 (arom.-*C*H), 125.1 (arom.-*C*H), 126.6 (arom.-*C*H), 127.5 (arom.-*C*H), 130.8 (arom.-*C*H), 130.8 (arom.-*C*H), 133.2 (C-3), 136.6 (arom.-*C*_ipso_), 136.6 (arom.-*C*_ipso_), 145.3 (*C*OO*p*NP), 155.6 (arom.-*C*_ipso_), 171.9 (arom.-*C*NO_2_). HR-MS (ESI, cation): calculated [C_21_H_23_NO_4_+Na^+^]: *m/z* = 376.15193; found [C_21_H_23_NO_4_+Na^+^]: *m/z* = 376.15211.

## Results

### Generation of fosmid libraries and phylogenetic analysis of hot spring and compost samples

Environmental samples were obtained from strong acidic and neutral hot spring water sediments and biofilms (pH ranging between 2.0 and 7.0, temperature from 60 to 62°C) from the São Miguel island, Azores, Portugal. In addition, a naturally heated lumber waste compost heap (63.3°C after 4 days) sample was obtained from a compost facility in Göttingen (Germany) (Table 1). High molecular weight DNA from both samples could be obtained and enabled the generation of a large-insert metagenomic library in the pCT3FK shuttle fosmid. The library comprised around 6048 single fosmid clones for the Azores samples and 1920 clones for the compost sample with each clone carrying approximately 35–40 kbp metagenomic inserts (Table 1). Phylogenetic analysis of the metagenomic DNA inserts was done by sequencing of PCR products obtained with degenerate primers which amplify bacterial and archaeal 16S rDNA sequences (Supplementary Table S1). This analysis showed a very low archaeal diversity in the hot spring water, with only representatives of the genera *Stygiolobus azoricus* DSM 6296 (99% sequence identity) and *Sulfolobus* species (e.g., *S. acidocaldarius* DSM 639 with 95% sequence identity) found. In the compost sample a more diverse representation of bacteria was observed with *Firmicutes* being the most highly represented phylum in which the majority of sequences were assigned to the orders *Bacillales* and *Clostridiales* (Table 2).

**Table 1:** **Overview of the constructed metagenomic libraries and the esterase screening results in *E. coli* EPI300 and *T. thermophilus* BL03**.

**Origin of metagenomic DNA**	**Sample information**	**Number of fosmid clones**	**Number of lipolytic clones (60°C)**	
			***E. coli* EPI300**	***T. thermophilus* BL03**
Hot springs (Azores Islands, Portugal)	AZ2 sediment, 60°C pH 6.0	384	1	1
	AZ3 sediment, 62°C, pH 2.0	5472	0	4
	AZ4 biofilms, 60°C, pH 7.0	192	0	0
Compost from lumber waste (Göttingen, Germany)	M12, heap II, 4th day, 63.3°C	1920	1	1
Summary		7968	2	6

**Table 2:** **Frequency of operational taxonomic units (OTUs) identified by 16S rDNA-analysis of the metagenomic samples**.

**Metagenomic sample**	**Kingdom**	**OTUs with highest similarity to phylum (e.g., most predominant families)**	**Number of sequences (frequency)**
Hot springs (water/sediments)	Archaea	*Crenarchaeota* (*Sulfolobaceae, Stygiolobus azoricus* DSM 6296 only)	5 (100.0%)
	Bacteria	*Proteobacteria* (*Enterobacteriaceae*, *Methylobacteriaceae, Acetobacteraceae, Sphingomonadaceae, Burkholderiaceae, Acidithiobacillaceae*)	19 (82.6%)
		*Bacteriodetes* (*Flavobacteriaceae*)	2 (8.7%)
		Others (*Cyanobacteria*)	2 (8.7%)
Compost (lumber waste)	Bacteria	*Firmicutes* (*Bacillaceae, Thermoanaerobacteriaceae, Alicyclobacillaceae, Paenibacillaceae, Clostridiaceae*)	208 (88.1%)
		*Actinobacteria* (*Streptosporangiaceae*)	17 (7.2%)
		*Proteobacteria* (*Enterobacteriaceae, Rhodobacteraceae Pseudomonadaceae, Myxococcaceae*)	8 (3.4%)
		Others (*Chloroflexi*, *Actinobacteria*)	3 (1.3%)

### Functional screening of fosmid libraries in *E. coli* and *T. thermophilus*

**Figure 1:**
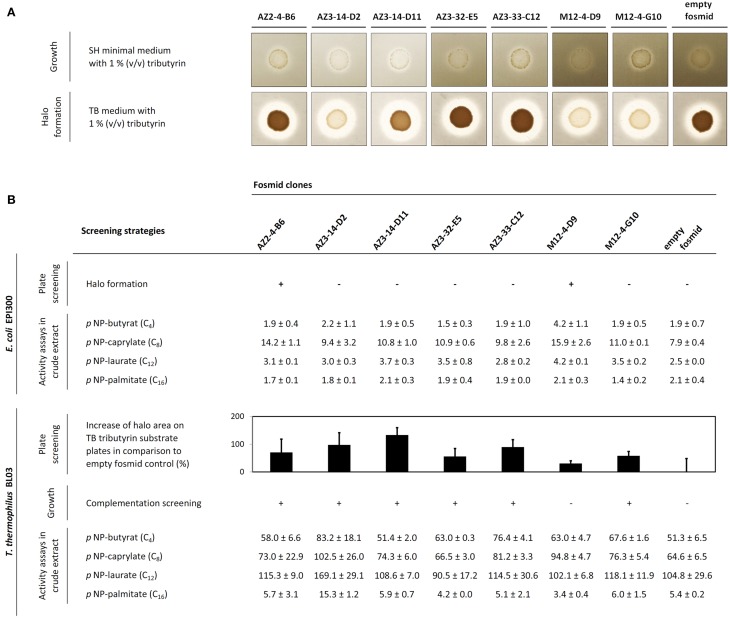
**(A)** Growth complementation screening results of *T. thermophilus* BL03 transformed with metagenomic fosmid DNA (upper scan picture) and corresponding tributyrase halo formation capabilities on substrate plates (lower scan picture). **(B)** Tributyrase activity of 8 single fosmids in *E. coli* EPI300 (upper part of the table) and *T. thermophilus* BL03 (below). In this representation, the increase of halo formation (black bars, halo area was calculated as described in the Materials and Methods section) on 1% (v/v) tributyrin substrate plates after 3 days of incubation at 60°C is shown (from triplicate measurements, *n* = 3; error bars indicate the standard deviation). In *T. thermophilus*, heterologous growth complementation results after 3 days of growth at 60°C on minimal medium are depicted. Comparative *p*NP-activity data from crude extracts (specific activity mU × mg^−1^) is shown for each single fosmid clone as average values from duplicate measurements (± standard deviation).

After screening of the generated libraries in *E. coli* using a plate assay at 60°C, only two halo-forming colonies were identified on tributyrin agar plates, one from the Azores sample (termed AZ2-4-B6) and one from the compost sample (M12-4-D9). In order to perform the screening in *T. thermophilus*, the BL03 strain was transformed with 83 fosmid pools, each prepared from 96 fosmid clones. The transformant pools obtained in this way were examined for their ability to grow on minimal medium plates supplemented with tributyrin. A total of 13 pools showed substantial growth on these plates (namely fosmid pools AZ2-4; AZ3-14; AZ3-25; AZ3-30; AZ3-32; AZ3-33; AZ3-37; AZ3-38; AZ3-47; AZ4-2; M12-3; M12-4; and M12-6). From these candidate pools, single fosmid transformations were performed in 96 well plate format. Again, growth on minimal medium was monitored, and positive clones were selected. After re-streaking them two more times to confirm the stability of their phenotype, six fosmid-containing *T. thermophilus* clones could be isolated from five different fosmid pools. They stably grew on minimal medium agar supplemented with tributyrin, and hydrolysis halos on tributyrin substrate plates were monitored in comparison with the BL03 strain carrying an empty fosmid (Figure 1A). In addition, the esterase activity of crude cell extracts obtained from the respective strains was measured with various *p*NP-substrates (Figure 1B). Almost all fosmid clones showed preferences for medium to short acyl chain fatty acid *p*NP-esters. Crude extract from the strain AZ3-14-D2 exhibited the highest activities of all crude extracts tested. This extract was most active over a broad range of *p*NP-substrates tested and its long-chain palmitate fatty acid hydrolysis was approximately three-fold higher than in the BL03 empty fosmid control. The two fosmids that conferred tributyrase activity in *E. coli*, AZ2-4-B6 and M12-4-D9, were also found to confer activity in *T. thermophilus* when tested in *p*NP-assays. Fosmid M12-4-D9 could not be identified by heterologous growth complementation screenings in *T. thermophilus* BL03, but showed higher activity on *p*NP-C_8_ compared to *E. coli*. In general, the *p*NP-substrate preference of the fosmid-encoded esterases for short-chain acyl esters was in agreement with the observed capabilities of halo formation on tributyrin agar plates.

### Sequencing and *in silico* analysis of the esterase activity-conferring fosmid inserts

The candidate fosmids were subjected to 454-sequencing and plasmids containing shotgun libraries of positive fosmids were sequenced with Sanger technology. The bioinformatic analysis of the fosmids is summarized in Table 3. Annotated α/β hydrolase 6 family proteins sharing high similarity to already characterized proteins were predicted from the sequence of two fosmids, namely M12-4-D9 and AZ2-4-B6. Not all positive fosmids from high-throughput *T. thermophilus* screenings could be completely assembled and sequenced (in average, 32.86 kb sequence information was obtained from each fosmid). The assembly data is summarized in Supplementary Table S2. Only in the case of two fosmids, AZ3-33-C12 (GenBank accession number KP892656) and M12-4-G10 (GenBank accession number KP892657) complete sequence assemblies where available for bioinformatic analysis. By motif search, the conserved GXSXG pentapeptide signature was identified in two of the predicted protein-encoding ORFs (EstA2 from fosmid AZ3-33-C12 and EstB1 from M12-4-G10). Both candidates showed no or only weak sequence similarity to lipolytic enzymes. Pfam prediction did not reveal any esterase/lipase-encoding function. For fosmid clones AZ3-32-E5, AZ3-14-D2, and AZ3-14-D11, sequence information was incomplete, therefore hampering further *in silico* analysis.

**Table 3:** **Overview of esterase-positive fosmids**.

**Fosmid clone**	**Functional screening positive in**	**Assembled sequence (kb) (no. of contigs)**	**Candidate ORF (contig)**	**Similarity search by blastp (closest *species*), additional information, characterized proteins**	**Predicted protein families and domains (Pfam)**
AZ2-4-B6^†^	*E. coli* and *T. thermophilus*	10.46 (2)	8c (contig 1)	Meta-fission product hydrolase (*Dyella ginsengisoli* LA-4) accession ACH87186.1 with 71% amino acid identity, a 2-hydroxy-6-oxo-6-phenylhexa-2,4-dienoate hydrolase (Li et al., 2009), α/β-hydrolase (*Alicycliphilus denitrificans* BC)	α/β-hydrolase 6
AZ3-14-D2	*T. thermophilus*	34.71 (4)	2 (contig 2)	Acetyl-CoA acetyltransferase (*Metallosphaera sedula*) WP_012020361.1 with 84% identity	Thiolase, N- and C-terminus
AZ3-14-D11	*T. thermophilus*	32.68 (2)	n.d.	*Sulfolobus* species	n.d.
AZ3-32-E5	*T. thermophilus*	28.97 (3)	12 (contig 1)	Dipeptidyl aminopeptidase/acylaminoacyl-peptidase (*Sulfolobus islandicus* LAL14/1), YP_007865031.1. with 60% identity; shares similarities to α/β hydrolases, COesterases and putative esterases, predicted active sites: Ser^412^, Asp^491^ and His^523^	Peptidase_S9
AZ3-33-C12	*T. thermophilus*	39.46 (1)	2	Hypothetical protein (*Acidianus hospitalis* W1) YP_004458132.1 with 66% similarity, phospholipase A2/esterase (*Desulfurococcus kamchatkensis* 1221n) YP_002428819.1 with 40% sequence identity, APE_2325 (*Aeropyrum pernix* K1) NP_148539.1 with 48% identity	Archaeal PaREP1/PaREP8 family, not significant
M12-4-D9^‡^	*E. coli*	shotgun clone	1	Hypothetical protein (*Caldibacillus debilis*) and carboxylesterase Est30 (*Geobacillus stearothermophilus*), crystal structure available at PDB: 1TQH, accession Q06174.2 with 71% similarity (Liu et al., 2004)	α/β-hydrolase 6
M12-4-G10	*T. thermophilus*	50.87 (1)	18c	Hypothetical protein (*Sphingobium quisquiliarum* P25), EQB08083.1 with 40% sequence identity	Metallo-β-lactamase B superfamily, alkyl sulfatase, N- and C-terminus

† *Sequenced separately at Göttingen Genomics Laboratory (G_2_L), no assembly statistics available, fosmid backbone pCT3FK sequence not included*.

‡ *Identified from shotgun library in E. coli only*.

*n.d., no detectable candidate ORF*.

### Heterologous expression and preliminary characterization of two novel metagenomic esterases

**Figure 2:**
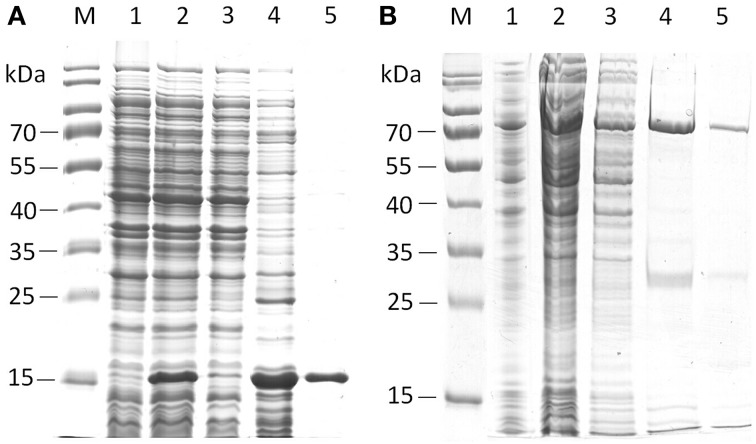
**SDS-PAGE analysis of the purification steps of his-tagged EstA2 (A) and EstB1 (B)**. Lanes M, protein ladder; Lanes 1, empty vector control; lanes 2, crude lysate; lanes 3, flow-through; Lanes 4–5, eluate fractions of target proteins. Predicted sizes of the proteins with C-terminal his-tag fusions are 18.7 kDa for EstA2 and 64.6 kDa for EstB1, respectively.

From all candidate esterase-encoding ORFs, we selected EstA2 and EstB1 for expression, purification and characterization using *T. thermophilus* as an expression host. Expression of these ORFs in pMK18 (de Grado et al., 1999) led to significant *p*NP-butyrate-cleaving activity (measured at 60°C). Further investigation revealed that short-chain acyl esters were the preferred substrates, such as *p*NP-butyrate, that was hydrolyzed most optimally around 80–85°C and pH 8.0. EstA2 (encoded on fosmid AZ3-33-C12) resulted in a significant activity increase in the extracellular fraction, resulting in a 1.54-fold induction of activity, while EstB1 showed a 1.53-fold increased activity level compared to 338.4 ± 4.8 mU/mg protein in BL03 with the empty pMK18 vector. However, no recombinant protein band could be detected by SDS-PAGE analysis. We then attempted to produce C-terminally tagged versions of both proteins in *E. coli* by using the pET21a(+) expression vector. For both proteins, purification using heat treatment followed by IMAC gravity flow columns resulted in functional protein preparations (Figure 2).

**Figure 3:**
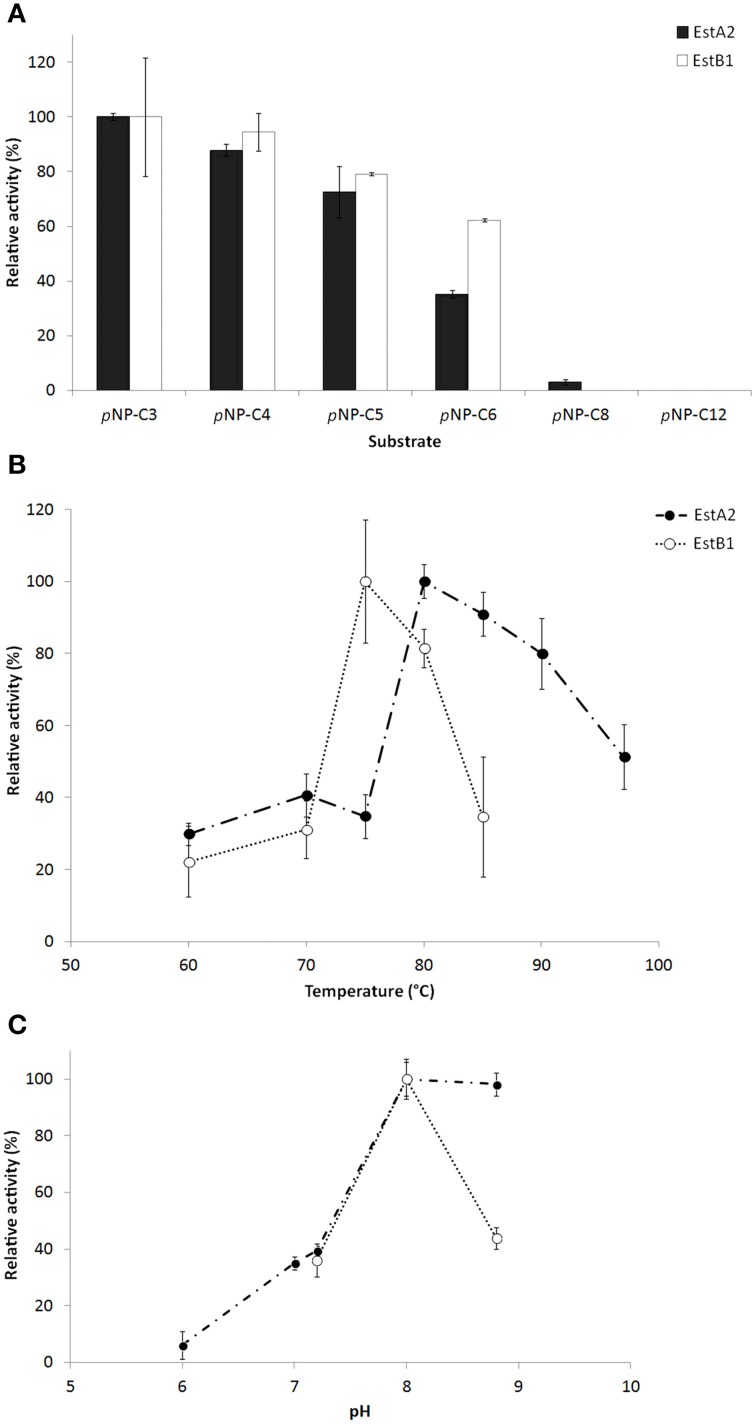
**Characterization of the purified esterase-active proteins EstA2 and EstB1. (A)** Substrate specificity of various acyl chain length *p*NP-esters. **(B)** Temperature and **(C)** pH optimum. The assays were performed in 50 mM phosphate buffer (pH 6–7) or 50 mM Tris-HCl buffer (pH 7.2–8.8) under optimal activity parameters (80°C for EstA2 and 75°C for EstB1, respectively) against 1.25 mM *p*NP-butyrate. 0.065–1.3 μg of purified enzyme was used for the assays. Data represents average values and standard deviations (error bars).

The characterization of the recombinant enzymes revealed similar substrate preferences for short acyl chain substrates (*p*NP-C_3_ > C_4_ > C_5_ > C_6_ > C_8_) as the ones observed in *T. thermophilus*, while activities on long-chain substrates (over C_10_) were comparably low (Figure 3). Under the assay conditions used here, optimum activity of EstA2 was observed around 80°C and pH 8.0, while EstB1 was most active at 75°C and pH 8.0. The specific activities of both enzymes on *para*-nitrophenyl butyrate were 44.05 ± 2.06 U/mg protein for EstA2 and 4.13 ± 0.71 U/mg for EstB1. The influence of several additives, NaCl, KCl, CaCl_2_, MgCl_2_, EDTA, and PMSF at 1–10 mM final concentration was negligible. Our data suggests both proteins to be esterases rather than lipases due to their characteristic substrate specificities.

EstA2 and EstB1 were tested toward various racemic mixtures of a selection of *p*NP-esters (Table 4). Activity was measured over a wide range of temperatures (60–80°C) to overcome constraints by autohydrolysis of the substrates at elevated temperatures (Chow et al., 2012; López et al., 2014). In general, the activities measured were comparably low (mU range). The highest activities under the conditions tested were found with the 4-nitrophenolates of-Naproxen and Ibuprofen. No activity could be detected with the *p*NP-esters of indancarboxylic acid while 2-methyldecanoate and 6-methyl-2-(*ortho*-tolyl)hept-5-enoate were only hydrolyzed weakly in the presence of EstB1 at the highest temperature tested so far.

**Table 4:** **Specific activity (mU/mg) of EstA2 and EstB1 on *p*NP-esters at various temperatures**.

**Tested substrates (*p*NP-derivatives)**	**60°C**		**70°C**		**80°C**	
	**EstA2**	**EstB1**	**EstA2**	**EstB1**	**EstA2**	**EstB1**
2-Methyldecanoate	–	–	–	–	–	0.15 ± 0.04
Ibuprofen	–	1.11 ± 0.31	0.68 ± 0.42	0.63 ± 0.02	2.55 ± 1.89	0.76 ± 0.02
Naproxen	1.01 ± 0.16	1.54 ± 0.03	2.94 ± 0.34	1.29 ± 0.72	3.09 ± 0.07	0.27 ± 0.06
4-Nitrophenyl 6-methyl-2-(*ortho*-tolyl)hept-5-enoate	–	–	–	–	–	0.63 ± 0.00
Indancarboxylic acid	–	–	–	–	–	–

*Activity tests were performed in 850 μL of 50 mM Sørensen buffer containing 0.1% (w/v) gum arabic and 5 mM sodium deoxycholate (pH 8.0 at 60, 70, and 80°C, respectively), mixed with 120 μL of DMSO and 20 μL of 10 mM of the pNP-ester substrates. Within 10 μL residual volume, 0.4 μg of the enzymes were added to the assay, which was performed for 30 min. Absorbance at 400 nm was measured in cuvettes, enzyme-free samples were measured as reference (the molar extinction coefficient was determined as ε = 14,000 M^−1^ × cm^−1^)*.

### Classification of lipolytic enzymes

**Figure 4:**
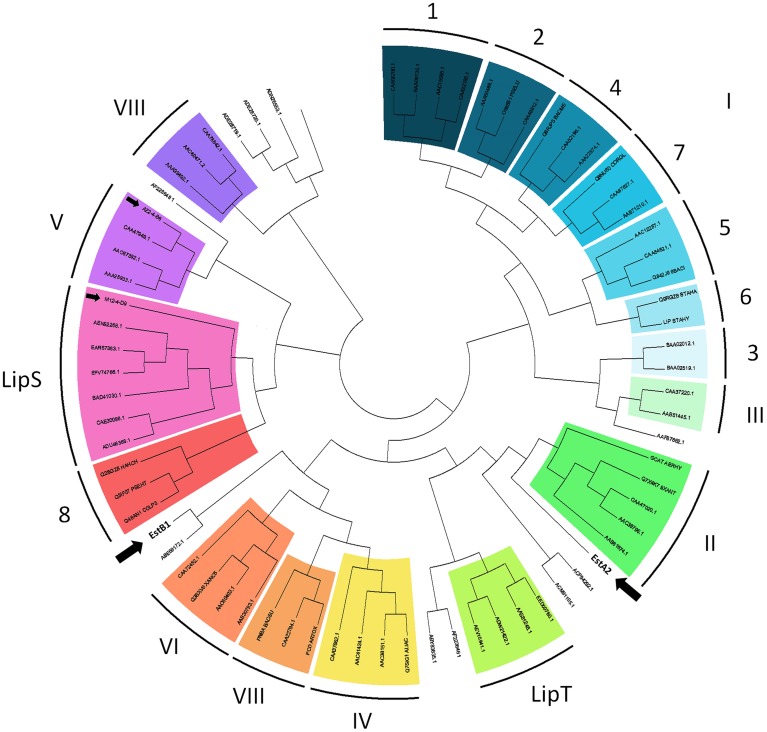
**Topologic view of a similarity tree of all lipolytic esterase and lipase protein families I to VIII (according to Arpigny and Jaeger, 1999), including the recently defined families LipS and LipT as well as classified, but still unknown protein families (Chow et al., 2012) and the new esterases from our functional metagenomic screenings (AZ2-4-B6, M12-4-D9, EstA2, EstB1)**. The data was generated from multiple sequence alignments of selected representative protein sequences (according to Chow et al., 2012) using the ClustalW algorithm (Boc et al., 2012). Tree reconstruction and visualization was performed with MEGA Version 5.2 (Tamura et al., 2011). The metagenomic proteins identified in this study are marked with black arrows. The length of the tree branches does not represent the phylogenetic distance between the protein sequences.

In order to classify the gene products identified in our metagenomic screenings, we performed multiple sequence alignments with known esterases and lipases from families I to VIII (Arpigny and Jaeger, 1999). Recently, the number of known lipolytic enzyme families has been extended by two new families, termed LipS and LipT (Chow et al., 2012). From the positive fosmids identified in *E. coli* (AZ2-4-B6 and M12-4-D9), both encoding α/β-hydrolase ORFs were identified as esterase class V and LipS family protein, respectively. The lipolytic enzymes discovered by using *T. thermophilus* for functional library screening, EstA2 and EstB1, could not be assigned to any known esterase/lipase family (Figure 4).

## Discussion

In the past, *E. coli* has been shown to be a good provider of interesting biocatalysts and enzymes from metagenomic screenings. Although it is clear that *E. coli* has restricted expression capabilities, it is the best developed and most commonly used host until today. Here, we show that using an additional host in functional screenings of metagenomic libraries can lead to the discovery of enzymes that would have remained hidden if only *E. coli* had been used.

How heterologous sequences are recognized and expressed into functional proteins by the transcriptional and translational machinery of an expression host is poorly understood. Factors influencing these processes, amongst other unknown factors, are: (i) toxicity of the foreign DNA and encoded proteins (Sorek et al., 2007), (ii) recognition of transcriptional signals (promoters) (Warren et al., 2008), (iii) ribosomal binding and translation initiation (Sørensen and Mortensen, 2005; Villegas and Kropinski, 2008), (iv) mRNA stability (Kudla et al., 2009), (v) codon usage (Sørensen and Mortensen, 2005), (vi) (post-translational) protein modification and secretion (Mergulhão et al., 2005). To overcome these restrictions, other screening hosts have been implemented in function-based metagenomic screening approaches in order to increase the detection frequency of active clones. First attempts using additional screening hosts involved *Streptomyces* and *Pseudomonas* species (Courtois et al., 2003; Martinez et al., 2004; Lussier et al., 2011). By the use of broad-host range vectors, the expression could recently be extended to other members of the *Proteobacteria*, i.e., the same taxon as *E. coli* (Aakvik et al., 2009; Craig et al., 2010; Kakirde et al., 2011), and to eukaryotic expression hosts (Damon et al., 2011; Kellner et al., 2011; Parachin and Gorwa-Grauslund, 2011). Our approach is distinguished from these “broad-host vectors” strategies by performing the library construction steps in the well-established *E. coli* system, followed by transfer of the metagenomic fosmids in the alternative host (Angelov et al., 2009). In order to facilitate the detection of esterase-encoding fosmid inserts, we used a selection-based strategy which relies on the complementation of growth of a custom, esterase-deficient strain of *T. thermophilus* (strain BL03, Leis et al., 2014). The BL03 strain is a multiple esterase deletion mutant which exhibits significantly reduced extracellular lipolytic activities compared with the parent strain HB27 and was shown to be severely impaired in growth on minimal medium supplemented with tributyrin (Leis et al., 2014). The growth could be regained via heterologous complementation when metagenomic DNA was expressed by the host. Low detection limits of classical phenotypic plate screenings (clearing zones around the colonies) could thus be overcome, as even weak gene expression levels yield enough metabolites to reconstitute growth. By implementing a high-throughput pooling strategy, approximately 8000 single fosmid clones yielded six single candidate fosmid clones, which represents a detection frequency that is comparable with screening results obtained from other metagenomics studies aimed at lipolytic activities from hot environments (Rhee et al., 2005; Chow et al., 2012). To our knowledge, this is the first report of identifying novel metagenome-borne esterases by the use of a screening organism other than traditional *E. coli*.

Metagenomic esterases having similarity to β-lactamase family proteins have been reported from various environmental or anthropogenic sources, e.g., leachate samples (Rashamuse et al., 2009), arctic soil (Yu et al., 2011), alkaline compost (Kim et al., 2010), drinking water and soil metagenomes (Elend et al., 2006). They share the conserved sequence motif common for class C β-lactamases (Ser-Xaa-Xaa-Lys, whereas Xaa is any amino acid) that is important for catalytic activity of esterases with similarity to penicillin-binding proteins (Petersen et al., 2001), and have been assigned to the less characterized family VIII carboxylesterases (Arpigny and Jaeger, 1999). EstB1 lacks this conserved sequence motif and does not belong to the class C β-lactamase protein family. Instead, it could be assigned to class B metallo-β-lactamase proteins (PF00753). This group comprises hydrolases acting on thioester (thioesterase) and sulfate ester (sulfatase) bonds. Furthermore, thioesterases have also been shown to convert *para*-nitrophenol esters (Shahi et al., 2006; Kotowska et al., 2009). EstB1 shared 30% sequence identity with SdsA1 from *Pseudomonas aeruginosa*, which is a characterized alkyl-sulfatase (Hagelueken et al., 2006). The other esterase-acting enzyme identified in our metagenomic studies was EstA2. It is most similar to hypothetical proteins from diverse archaeal origins. One characterized enzyme, to which EstA2 shows very weak similarity, is a phospholipase from the hyperthermophilic archaeon *Aeropyrum pernix* K1 encoded by ORF APE2325 (Wang et al., 2004). Also being a small protein with 18 kDa in size, its reported substrate spectrum and activity pattern is comparable to EstA2, as it also preferred short acyl chain *p*NP-substrates and was most active at elevated temperatures (above 80°C).

In this study we have demonstrated that *T. thermophilus* is an excellent host bacterium for the detection of novel metagenome-borne enzymes that could not readily have been detected by the use of *E. coli* or by *in silico* analysis. To our knowledge, this is the first report that metagenome-derived esterases could be identified in an expression host other than *E. coli*. The functional screening implementing *T. thermophilus* BL03 could uncover several lipolytic enzymes from underrepresented species and archaeal origin, and most strikingly, some of them could not be assigned to classical esterase and lipase enzyme families and therefore represent truly novel esterase-active proteins. The confirmation of predicted ORFs responsible for the lipolytic activity of the remaining fosmids will be addressed in the future. The use of complementation screens in *T. thermophilus* with shotgun subclones will help to identify more active genes that do not share any known sequence signatures at all.

### Conflict of interest statement

The authors declare that the research was conducted in the absence of any commercial or financial relationships that could be construed as a potential conflict of interest.

## References

[B1] Aakvik T. Degnes K. F. Dahlsrud R. Schmidt F. Dam R. Yu L.. (2009). A plasmid RK2-based broad-host-range cloning vector useful for transfer of metagenomic libraries to a variety of bacterial species. FEMS Microbiol. Lett. 296, 149–158. 10.1111/j.1574-6968.2009.01639.x 19459950

[B2] Altschul S. F. Gish W. Miller W. Myers E. W. Lipman D. J. (1990). Basic local alignment search tool. J. Mol. Biol. 215, 403–410. 10.1016/S0022-2836(05)80360-2 2231712

[B3] Amann R. I. Ludwig W. Schleifer K. H. (1995). Phylogenetic identification and *in situ* detection of individual microbial cells without cultivation. Microbiol. Rev. 59, 143–169. 7535888 10.1128/mr.59.1.143-169.1995 PMC239358

[B4] Angelov A. Mientus M. Liebl S. Liebl W. (2009). A two-host fosmid system for functional screening of (meta)genomic libraries from extreme thermophiles. Syst. Appl. Microbiol. 32, 177–185. 10.1016/j.syapm.2008.01.003 19285378

[B5] Arpigny J. L. Jaeger K. E. (1999). Bacterial lipolytic enzymes: classification and properties. Biochem. J. 343, 177–183. 10.1042/0264-6021:3430177 10493927 PMC1220539

[B6] Boc A. Diallo A. B. Makarenkov V. (2012). T-REX: a web server for inferring, validating and visualizing phylogenetic trees and networks. Nucleic Acid Res. 40, W573–W579. 10.1093/nar/gks485 22675075 PMC3394261

[B9] Chow J. Kovacic F. Dall Antonia Y. Krauss U. Fersini F. Schmeisser C.. (2012). The metagenome-derived enzymes LipS and LipT increase the diversity of known lipases. PLoS ONE 7:e47665. 10.1371/journal.pone.0047665 23112831 PMC3480424

[B10] Cole J. R. Wang Q. Cardenas E. Fish J. Chai B. Farris R. J.. (2009). The Ribosomal Database Project: improved alignments and new tools for rRNA analysis. Nucleic Acid. Res. 37, D141–D145. 10.1093/nar/gkn879 19004872 PMC2686447

[B11] Courtois S. Cappellano C. M. Ball M. Francou F.-X. Normand P. Helynck G.. (2003). Recombinant environmental libraries provide access to microbial diversity for drug discovery from natural products. Appl. Environ. Microbiol. 69, 49–55. 10.1128/AEM.69.1.49-55.2003 12513976 PMC152451

[B12] Craig J. W. Chang F.-Y. Kim J. H. Obiajulu S. C. Brady S. F. (2010). Expanding small-molecule functional metagenomics through parallel screening of broad-host-range cosmid environmental DNA libraries in diverse proteobacteria. Appl. Environ. Microbiol. 76, 1633–1641. 10.1128/AEM.02169-09 20081001 PMC2832356

[B13] Damon C. Vallon L. Zimmermann S. Haider M. Z. Galeote V. Dequin S.. (2011). A novel fungal family of oligopeptide transporters identified by functional metatranscriptomics of soil eukaryotes. ISME J. 5, 1871–1880. 10.1038/ismej.2011.67 21654847 PMC3223307

[B14] de Grado M. Castán P. Berenguer J. (1999). A high-transformation-efficiency cloning vector for *Thermus thermophilus*. Plasmid 42, 241–245. 10.1006/plas.1999.1427 10545266

[B15] Elend C. Schmeisser C. Leggewie C. Babiak P. Carballeira J. D. Steele H. L.. (2006). Isolation and biochemical characterization of two novel metagenome-derived esterases. Appl. Environ. Microbiol. 72, 3637–3645. 10.1128/AEM.72.5.3637-3645.2006 16672512 PMC1472341

[B16] Franken B. Jaeger K.-E. Pietruszka J. (2010). Protocols to screen for enantioselective lipases, in Handbook of Hydrocarbon and Lipid Microbiology, ed Timmes K. N. (Berlin-Heidelberg: Springer), 4581–4586.

[B18] Gabor E. M. Alkema W. B. L. Janssen D. B. (2004). Quantifying the accessibility of the metagenome by random expression cloning techniques. Environ. Microbiol. 6, 879–886. 10.1111/j.1462-2920.2004.00640.x 15305913

[B19] Gaich T. Mulzer J. (2009). Total synthesis of (-)-Penifulvin A, an insecticide with a dioxafenestrane skeleton. J. Am. Chem. Soc. 131, 452–453. 10.1021/ja8083048 19140787

[B20] Hagelueken G. Adams T. M. Wiehlmann L. Widow U. Kolmar H. Tümmler B.. (2006). The crystal structure of SdsA1, an alkylsulfatase from *Pseudomonas aeruginosa*, defines a third class of sulfatases. Proc. Natl. Acad. Sci. U.S.A. 103, 7631–7636. 10.1073/pnas.0510501103 16684886 PMC1472496

[B21] Handelsman J. Rondon M. R. Brady S. F. Clardy J. Goodman R. M. (1998). Molecular biological access to the chemistry of unknown soil microbes: a new frontier for natural products. Chem. Biol. 5, R245–R249. 10.1016/S1074-5521(98)90108-9 9818143

[B22] Jenney F. E. Adams M. W. W. (2008). The impact of extremophiles on structural genomics (and vice versa). Extremophiles 12, 39–50. 10.1007/s00792-007-0087-9 17563834

[B23] Kakirde K. S. Wild J. Godiska R. Mead D. A. Wiggins A. G. Goodman R. M.. (2011). Gram negative shuttle BAC vector for heterologous expression of metagenomic libraries. Gene 475, 57–62. 10.1016/j.gene.2010.11.004 21112378 PMC3058121

[B24] Kellner H. Luis P. Portetelle D. Vandenbol M. (2011). Screening of a soil metatranscriptomic library by functional complementation of *Saccharomyces cerevisiae* mutants. Microbiol. Res. 166, 360–368. 10.1016/j.micres.2010.07.006 20869217

[B25] Kim Y. H. Kwon E. J. Kim S. K. Jeong Y. S. Kim J. Yun H. D.. (2010). Molecular cloning and characterization of a novel family VIII alkaline esterase from a compost metagenomic library. Biochem. Biophys. Res. Comm. 393, 45–49. 10.1016/j.bbrc.2010.01.070 20097165

[B26] Kotowska M. Pawlik K. Smulczyk-Krawczyszyn A. Bartosz-Bechowski H. Kuczek K. (2009). Type II Thioesterase ScoT, Associated with *Streptomyces coelicolor* A3(2) modular polyketide synthase Cpk, hydrolyzes acyl residues and has a preference for propionate. Appl. Environ. Microbiol. 75, 887–896. 10.1128/AEM.01371-08 19074611 PMC2643599

[B27] Kudla G. Murray A. W. Tollervey D. Plotkin J. B. (2009). Coding-sequence determinants of gene expression in *Escherichia coli*. Science 324, 255–258. 10.1126/science.1170160 19359587 PMC3902468

[B28] Laemmli U. K. (1970). Cleavage of structural proteins during the assembly of the head of bacteriophage T4. Nature 227, 680–685. 10.1038/227680a0 5432063

[B29] Lane D. J. (1991). 16S/23S rRNA sequencing, in Nucleic Acid Techniques in Bacterial Systematics, eds Stackebrandt E. Goodfellow M. (Chichester, NY: Wiley), 115–175.

[B30] Leis B. Angelov A. Liebl W. (2013). Screening and expression of genes from metagenomes. Adv. Appl. Microbiol. 83, 1–68. 10.1016/B978-0-12-407678-5.00001-5 23651593

[B31] Leis B. Angelov A. Li H. Liebl W. (2014). Genetic analysis of lipolytic activities in *Thermus thermophilus* HB27. J. Biotechnol. 191, 150–157. 10.1016/j.jbiotec.2014.07.448 25102235

[B32] Li A. Qu Y. Zhou J. Ma F. (2009). Enzyme-substrate interaction and characterization of a 2,3-dihydroxybiphenyl 1,2-dioxygenase from *Dyella ginsengisoli* LA-4. FEMS Microbiol. Lett. 292, 231–239. 10.1111/j.1574-6968.2009.01487.x 19187202

[B33] Liebl W. (2011). Metagenomics, in Encyclopedia of Geobiology, Encyclopedia of Earth Sciences Series, eds Reitner J. Thiel V. (Dordrecht, NL: Springer), 553–558.

[B34] Liebl W. Angelov A. Juergensen J. Chow J. Loeschcke A. Drepper T.. (2014). Alternative hosts for functional (meta)genome analysis. Appl. Microbiol. Biotechnol. 98, 8099–8109. 10.1007/s00253-014-5961-7 25091044

[B35] Liu P. Wang Y.-F. Ewis H. E. Abdelal A. T. Lu C.-D. Harrison R. W.. (2004). Covalent reaction intermediate revealed in crystal structure of the *Geobacillus stearothermophilus* carboxylesterase Est30. J. Mol. Biol. 342, 551–561. 10.1016/j.jmb.2004.06.069 15327954

[B36] López G. Chow J. Bongen P. Lauinger B. Pietruszka J. Streit W. R.. (2014). A novel thermostable esterase from *Acidicaldus* sp. strain USBA-GBX-499 with enantioselectivity isolated from an acidic hot spring of Colombian Andes. Appl. Microbiol. Biotechnol. 98, 8603–8616. 10.1007/s00253-014-5775-7 24818691

[B37] Ludwig W. Strunk O. Westram R. Richter L. Meier H. Yadhukumar. (2004). ARB: a software environment for sequence data. Nucleic Acids Res. 32, 1363–1371. 10.1093/nar/gkh293 14985472 PMC390282

[B38] Lussier F.-X. Chambenoit O. Côté A. Hupé J.-F. Denis F. Juteau P.. (2011). Construction and functional screening of a metagenomic library using a T7 RNA polymerase-based expression cosmid vector. J. Ind. Microbiol. Biotechnol. 38, 1321–1328. 10.1007/s10295-010-0915-2 21108039

[B39] Marchler-Bauer A. Lu S. Anderson J. B. Chitsaz F. Derbyshire M. K. DeWeese-Scott C.. (2011). CDD: a Conserved Domain Database for the functional annotation of proteins. Nucleic Acids Res. 39, D225–D229. 10.1093/nar/gkq1189 21109532 PMC3013737

[B40] Martinez A. Kolvek S. J. Yip C. L. T. Hopke J. Brown K. A. MacNeil I. A.. (2004). Genetically modified bacterial strains and novel bacterial artificial chromosome shuttle vectors for constructing environmental libraries and detecting heterologous natural products in multiple expression hosts. Appl. Environ. Microbiol. 70, 2452–2463. 10.1128/AEM.70.4.2452-2463.2004 15066844 PMC383137

[B41] Mergulhão F. J. M. Summers D. K. Monteiro G. A. (2005). Recombinant protein secretion in *Escherichia coli*. Biotechnol. Adv. 23, 177–202. 10.1016/j.biotechadv.2004.11.003 15763404

[B42] Overbeek R. Begley T. Butler R. M. Choudhuri J. V. Chuang H.-Y. Cohoon M. (2005). The subsystems approach to genome annotation and its use in the project to annotate 1000 genomes. Nucleic Acids Res. 33, 5691–5702. 10.1093/nar/gki866 16214803 PMC1251668

[B43] Parachin N. S. Gorwa-Grauslund M. F. (2011). Isolation of xylose isomerases by sequence- and function-based screening from a soil metagenomic library. Biotechnol. Biofuels 4:9. 10.1186/1754-6834-4-9 21545702 PMC3113934

[B44] Petersen E. I. Valinger G. Sölkner B. Stubenrauch G. Schwab H. (2001). A novel esterase from *Burkholderia gladioli* which shows high deacetylation activity on cephalosporins is related to beta-lactamases and DD-peptidases. J. Biotechnol. 89, 11–25. 10.1016/S0168-1656(01)00284-X 11472796

[B45] Petersen T. N. Brunak S. Heijne G. von Nielsen H. (2011). SignalP 4.0: discriminating signal peptides from transmembrane regions. Nat. Methods 8, 785–786. 10.1038/nmeth.1701 21959131

[B46] Pietruszka J. Simon R. C. Kruska F. Braun M. (2009). Dynamic enzymatic kinetic resolution of methyl 2,3-dihydro-1H-indene-1-carboxylate. Eur. J. Org. Chem. 2009, 6217–6224 10.1002/ejoc.200901025

[B47] Punta M. Coggill P. C. Eberhardt R. Y. Mistry J. Tate J. Boursnell C.. (2012). The Pfam protein families database. Nucleic Acids Res. 40, D290–D301. 10.1093/nar/gkr1065 22127870 PMC3245129

[B48] Ramírez-Arcos S. Fernández-Herrero L. A. Marín I. Berenguer J. (1998). Anaerobic growth, a property horizontally transferred by an Hfr-like mechanism among extreme thermophiles. J. Bacteriol. 180, 3137–3143. 9620963 10.1128/jb.180.12.3137-3143.1998 PMC107814

[B49] Rashamuse K. Magomani V. Ronneburg T. Brady D. (2009). A novel family VIII carboxylesterase derived from a leachate metagenome library exhibits promiscuous beta-lactamase activity on nitrocefin. Appl. Microbiol. Biotechnol. 83, 491–500. 10.1007/s00253-009-1895-x 19190902

[B50] Reetz M. T. Prasad S. Carballeira J. D. Gumulya Y. Bocola M. (2010). Iterative saturation mutagenesis accelerates laboratory evolution of enzyme stereoselectivity: rigorous comparasion with traditional methods. J. Am. Chem. Soc. 132, 9144–9152. 10.1021/ja1030479 20536132

[B51] Reetz M. T. Zonta A. Schimossek K. Jaeger K.-E. Liebeton K. (1997). Creation of enantioselective biocatalysts for organic chemistry by *in vitro* evolution. Angew. Chem. Int. Ed. Engl. 36, 2830–2832 10.1002/anie.199728301

[B52] Rhee J. K. Ahn D. G. Kim Y. G. Oh J. W. (2005). New thermophilic and thermostable esterase with sequence similarity to the hormone-sensitive lipase family, cloned from a metagenomic library. Appl. Environ. Microbiol. 71, 817–825. 10.1128/AEM.71.2.817-825.2005 15691936 PMC546692

[B53] Sandström A. G. Wikmark Y. Engström K. Nyhlén J. Bäckvall J.-E. (2012). Combinatorial reshaping of the *Candida Antarctica* lipase A substrate pocket for enantioselectivity using an extremely condensed library. Proc. Natl. Acad. Sci. U.S.A. 109, 78–83. 10.1073/pnas.1111537108 22178758 PMC3252943

[B54] Satokari R. M. Vaughan E. E. Akkermans A. D. Saarela M. de Vos W. M. (2001). Bifidobacterial diversity in human feces detected by genus-specific PCR and denaturing gradient gel electrophoresis. Appl. Environ. Microbiol. 67, 504–513. 10.1128/AEM.67.2.504-513.2001 11157210 PMC92614

[B55] Shahi P. Kumar I. Sharma R. Sanger S. Jolly R. S. (2006). Characterization of a novel long-chain acyl-CoA thioesterase from *Alcaligenes faecalis*. FEBS J. 273, 2374–2387. 10.1111/j.1742-4658.2006.05244.x 16704412

[B57] Sorek R. Zhu Y. Creevey C. J. Francino M. P. Bork P. Rubin E. M. (2007). Genome-wide experimental determination of barriers to horizontal gene transfer. Science 318, 1449–1452. 10.1126/science.1147112 17947550

[B58] Sørensen H. P. Mortensen K. K. (2005). Advanced genetic strategies for recombinant protein expression in *Escherichia coli*. J. Biotechnol. 115, 113–128. 10.1016/j.jbiotec.2004.08.004 15607230

[B59] Steele H. L. Jaeger K.-E. Daniel R. Streit W. R. (2009). Advances in recovery of novel biocatalysts from metagenomes. J. Mol. Microbiol. Biotechnol. 16, 25–37. 10.1159/000142892 18957860

[B60] Tamura K. Peterson D. Peterson N. Stecher G. Nei M. Kumar S. (2011). MEGA5: molecular evolutionary genetics analysis using maximum likelihood, evolutionary distance, and maximum parsimony methods. Mol. Biol. Evol. 28, 2731–2739. 10.1093/molbev/msr121 21546353 PMC3203626

[B61] Torsvik V. Goksøyr J. Daae F. L. (1990). High diversity in DNA of soil bacteria. Appl. Environ. Microbiol. 56, 782–787. 2317046 10.1128/aem.56.3.782-787.1990 PMC183421

[B62] Villegas A. Kropinski A. M. (2008). An analysis of initiation codon utilization in the Domain Bacteria—concerns about the quality of bacterial genome annotation. Microbiology 154, 2559–2661. 10.1099/mic.0.2008/021360-0 18757789

[B63] Wang B. Lu D. Gao R. Yang Z. Cao S. Feng Y. (2004). A novel phospholipase A2/esterase from hyperthermophilic archaeon *Aeropyrum pernix* K1. Protein Expr. Purif. 35, 199–205. 10.1016/j.pep.2004.01.010 15135393

[B64] Warren R. L. Freeman J. D. Levesque R. C. Smailus D. E. Flibotte S. Holt R. A. (2008). Transcription of foreign DNA in *Escherichia coli*. Genome Res. 18, 1798–1805. 10.1101/gr.080358.108 18701636 PMC2577866

[B65] Wu Q. Soni P. Reetz M. T. (2013). Laboratory evolution of enantiocomplementary *Candida antarctica* lipase B mutants with broad substrate scope. J. Am. Chem. Soc. 135, 1872–1881. 10.1021/ja310455t 23301759

[B66] Yu E. Y. Kwon M.-A. Lee M. Oh J. Y. Choi J.-E. Lee J. Y.. (2011). Isolation and characterization of cold-active family VIII esterases from an arctic soil metagenome. Appl. Microbiol. Biotechnol. 90, 573–581. 10.1007/s00253-011-3132-7 21318360

