# Supplementary material for: Identification of novel esterase-active enzymes from hot environments by use of the host bacterium Thermus thermophilus
Source: Front Microbiol. 2015 Apr 8;6:275. doi: 10.3389/fmicb.2015.00275 (PMC4389547; doi:10.3389/fmicb.2015.00275)
Supplement: Supplementary file 1 [file DataSheet1.DOC]

**Supplementary Table S1: Oligonucleotides used in this study.**

|  | |  | | |
| --- | --- | --- | --- | --- |
| **Primer name** | **5’→3’ sequence** | | **Used for** |  |
|  |
| 616Valt | AGAGTTTGATYMTGGCTCAG | | Amplification of bacterial 16S rDNA (Lane, 1991; Satokari *et al.*, 2001) |  |
| 100K | GGTTACCTTGTTACGACTT | |  |
| AC165 | TATAGCGGCCGCATTCYGKTTGATCCYGSG | | Amplification of archaeal 16S rDNA (Department of Microbiology, Technische Universität München) |  |
| AC1601 | GGGCGGTGTGTGCAA | |  |
| EstA2_for | ATTAAGGAGGTGTGAGGCATATGGTTCAGAGTAAAAAGAT | | Cloning of EstA2 encoding sequence in expression vectors |  |
| EstA2_rev | GGCTCGAGTTTTTCAGCAAGTTATCTCTTTTCTAAAATCT | |  |
| EstB1_for | ATTAAGGAGGTGTGAGGCATATGCCAATCCCGGAACCACT | | Cloning of EstB1 encoding sequence in expression vectors |  |
| EstB1_rev | GGCTCGAGTTTTTCAGCAAGCTACCGCAGTGCGAGTGCCGG | |  |
| EstA2_PHO_F | CAAAGATTTTAGAAAAGAGACACCACCACCACCACCACTAACTTGCTGAAAAACTCGA | | Site directed mutagenesis primers for the insertion of a poly-His tag at the C-terminus of EstA2 and EstB1 |  |
| EstB1_PHO_F | TCCCGGCACTCGCACTGCGGCACCACCACCACCACCACTAGCTTGCTGAAAAACTCGA | |  |
| pMK18_RBS_for | CATCCGGAAGATCTGGCGGCGAATTCGTAATCATGTCATA | | Primer pair for amplification of pMK18 vector, introduction of a RBS site upstream of the target ORFs |  |
| pMK18_RBS_rev | ATGCCTCACACCTCCTTAATAGTCGACCTGCAGGCATGCA | |  |
| Est_rev | GCCGCCAGATCTTCCGGATGGCTCGAGT | | Reverse primer for cloning metagenomic esterases in pMK18 vector |  |
|  | |  | | |

| **Supplementary Table S2: Assembly statistics of 454-sequencing reads.** | | | | | | | | |  |  |  |
| --- | --- | --- | --- | --- | --- | --- | --- | --- | --- | --- | --- |
|  |  |  |  |  |  |  |  |  |  |  |  |
| **Fosmid clone** |  | **AZ3-32-E5** |  | **AZ3-33-C12** |  | **M12-4-G10** |  | **AZ3-14-D2** |  | **AZ3-14-D11** |  |
|  |  |  |  |  |  |  |  |  |  |  |  |
| No. aligned reads (%) |  | 31,312 (98.07%) |  | 17,308 (95.83%) |  | 12,611 (97.26%) |  | 12,912 (96.97%) |  | 12,006 (95.55%) |  |
| No. aligned bases (%) |  | 15,347,368 (99.50%) |  | 8,225,536 (96.98%) |  | 6,033,363 (99.09%) |  | 6,255,430 (98.43%) |  | 5,735,887 (96.80%) |  |
| read error (%) |  | 73,898 (0.48%) |  | 43,525 (0.53%) |  | 26,038 (0.43%) |  | 30,918 (0.49%) |  | 27,697 (0.48%) |  |
|  |  |  |  |  |  |  |  |  |  |  |  |
| assembled reads |  | 30,804 |  | 16,284 |  | 12,518 |  | 11,909 |  | 11,000 |  |
| partial reads |  | 508 |  | 1,024 |  | 93 |  | 1,003 |  | 1,006 |  |
| singletons |  | 76 |  | 428 |  | 94 |  | 122 |  | 317 |  |
| repeats |  | 0 |  | 0 |  | 0 |  | 0 |  | 0 |  |
| outlier |  | 40 |  | 26 |  | 2 |  | 20 |  | 11 |  |
| too short reads |  | 500 |  | 300 |  | 259 |  | 262 |  | 231 |  |
|  |  |  |  |  |  |  |  |  |  |  |  |
| No. contigs |  | 3 |  | 1 |  | 1 |  | 4 |  | 2 |  |
| No. bases |  | 28,967 |  | 39,462 |  | 50,871 |  | 34,714 |  | 32,684 |  |
|  |  |  |  |  |  |  |  |  |  |  |  |
| average contig size |  | 9,655 |  | 39,462 |  | 50,871 |  | 8,678 |  | 16,342 |  |
| largest contig size |  | 16,376 |  | 39,462 |  | 50,871 |  | 12,694 |  | 21,047 |  |
|  |  |  |  |  |  |  |  |  |  |  |  |
|  |  |  |  |  |  |  |  |  |  |  |  |
| AZ2-4-B6 was sequenced at G_2_L sequencing laboratory in Göttingen, Germany. No information about assembly statistics available. | | | | | | | | | | | |
| M12-4-D9 active ORF was identified by generation of a shotgun library from fosmid DNA. | | | | | | | | |  |  |  |
